# Knot Energy, Complexity, and Mobility of Knotted Polymers

**DOI:** 10.1038/s41598-017-12461-w

**Published:** 2017-10-17

**Authors:** Fernando Vargas–Lara, Ahmed M. Hassan, Marc L. Mansfield, Jack F. Douglas

**Affiliations:** 1 Materials Science and Engineering Division, National Institute of Standards and Technology, Gaithersburg, MD 20899 USA; 2 Department of Computer Science and Electrical Engineering, University of Missouri–Kansas City, Kansas City, MO 64110 USA; 3 Bingham Research Center, Utah State University, Vernal, UT 84078 USA

## Abstract

The Coulomb energy *E*_C_ is defined by the energy required to charge a conductive object and scales inversely to the self–capacity *C*, a basic measure of object size and shape. It is known that *C* is minimized for a sphere for all objects having the same volume, and that *C* increases as the symmetry of an object is reduced at fixed volume. Mathematically similar energy functionals have been related to the average knot crossing number 〈m〉, a natural measure of knot complexity and, correspondingly, we find *E*_C_ to be directly related to 〈m〉 of knotted DNA. To establish this relation, we employ molecular dynamics simulations to generate knotted polymeric configurations having different length and stiffness, and minimum knot crossing number values *m* for a wide class of knot types relevant to the real DNA. We then compute *E*_C_ for all these knotted polymers using the program ZENO and find that the average Coulomb energy 〈*E*_C_〉 is directly proportional to 〈m〉. Finally, we calculate estimates of the ratio of the hydrodynamic radius, radius of gyration, and the intrinsic viscosity of semi–flexible knotted polymers in comparison to the linear polymeric chains since these ratios should be useful in characterizing knotted polymers experimentally.

## Introduction

Experiments have shown a remarkable correlation between the migration speed of knotted DNA in gel electrophoresis and average knot crossing number, the number of places where a knotted polymer crosses itself when projected onto a surface^1,2^. An early study indicated a correlation between DNA mobility in gels with the minimum knot crossing number *m* ^1^, but Stasiak *et al*.^2^ later found a better correlation of knotted DNA electrophoretic mobility with the crossing number averaged over all polymer conformations, 〈*m*〉. The minimum crossing number *m* is a topological invariant found in knot classification^3,4^, but numerical studies have established that the configurationally averaged 〈*m*〉 varies with chain length, chain stiffness, and the strength of the excluded volume interaction^5^. These previous experimental and computational studies raise questions^6^ about which property of knotted polymers dominates the DNA separation process by electrophoresis and about the accurate computation of traslational friction coefficient of knotted polymers for comparison to both sedimentation and electrophoresis measurements.

The utilization of energy functionals to classify object shape has a long history. For example, it has been appreciated since the time of the ancient Greeks that a sphere has the minimum surface area of all the objects having a given volume and it is common to classify particle shape in terms of the relative surface area of a particle to a sphere having the same volume, i.e., “sphericity”^7,8^. In many applications, minimum surface area directly corresponds to a minimizing energy, e.g., the interfacial energy of a droplet defines an “energy functional” and fluid droplets of ordinary fluids are accordingly spherical in order to minimize their interfacial energy and thus their surface area. Poincaré first proved that the electrostatic capacity *C* of a finite volume region is similarly minimized by a spherical shape in connection with his study of the rotation of liquid droplets^9^, and Szegö later proved this “isoperimetric” relation rigorously^10^. Pólya and Szegö embarked on a ambitious program of object shape classification in terms of *C* and other energy functionals related to the Laplace’s equation (hydrodynamic virtual mass, magnetic, and electric polarizability)^11–13^. In each case, a scalar energy functional can be defined which is minimized by a sphere for all objects having a finite volume. Garboczi *et al*. have illustrated the dependence of these functionals on shape in the case of ellipsoidal particles as part of a study of the shape dependence of the percolation threshold of overlapping objects^14^.

Capacity is an especially important energy functional because of its many physical applications. It governs the rate of heat transfer from an object based on Newton’s law of cooling, the rate of diffusion–limited reactions, scattering lengths in acoustic and quantum theory, as well as its well known interpretation in electrostatics^12,14,15^. Historically, the use of this functional for shape classification has been limited by the difficulty of calculating *C* for complicated shaped objects. For example, the calculation of *C* for a cube is still an unsolved analytic problem^12^. Numerical path–integration methods, however, have recently allowed the accurate numerical computation of *C* for regions having essentially arbitrary complexity^16,17^. Tests against exactly solvable cases show these path–integral calculations provide accurate estimates of *C* for reasonable computational times^15,16^. We are now in position of calculating *C* for the purpose of shape and topology classification and we are interested in the present paper in classifying knotted polymers in terms of *C*. This enables an extension of the shape classification program initiated by Pólya and Szegö to describe the topological properties of objects using basic energy functionals^12^.

Since *C* is important in our discussion below, it is worth recalling its mathematical definition. Consider a conductive object Γ having a fixed charge *q* that is distributed at the equilibrium on the object surface ∂Γ. The “Coulomb energy” *E*_C_ of the equilibrium charge distribution on a conductive object equals,

1 $${E}_{{\rm{C}}}=\frac{{q}^{2}}{8\,\pi \,\varepsilon \,C},$$

where *ε* is the dielectric constant of the medium in which the charged object is placed. The “Coulomb constant” (1/4 *π ε*) in this relationship defines the proportionality factor of the Coulomb potential, and following mathematical conventions, we take this quantity, along with *q*, to be equal 1 so that *E*_C_ = 1/2 *C*. The Coulomb energy is familiar in a physical chemistry context as the basis of the Born theory for calculating ion solvation energies^18,19^, where ions are modeled as charged spheres. Duhr and Brown^20^ have argued that the solvation energy of duplex DNA can be estimated from an extended Born model where the effective radius of the charged DNA molecule is estimated from the DNA hydrodynamic radius, which as we will see below is related to *C*.

The Coulomb energy can also be defined as an energy functional (Kelvin’s principle)^12,17^,

2 $${E}_{{\rm{C}}}={\int }_{{\rm{\partial }}{\rm{\Gamma }}}dR{\int }_{{\rm{\partial }}{\rm{\Gamma }}}d{R}^{{\rm{^{\prime} }}}\frac{\rho (R)\,\rho ({R}^{{\rm{^{\prime} }}})}{|R-{R}^{{\rm{^{\prime} }}}|},$$

which is minimized by the equilibrium charge density *ρ*(*R*) normalized so that, ∫_∂Γ_ *ρ*(*R*)*dR* = 1, where *R* is a point on ∂Γ. Alternatively, *C* can also be defined through the minimum energy of the potential field gradient exterior to the conductive object (Dirichlet’s principle). This complementary definition of *C* shows that this equation describes the asymptotic decay of the solution of the Laplace’s equation on the exterior of a region at distances far from the object where the solution of Laplace’s equation is constant on the boundary. This is the classic exterior Dirichlet problem^17^. This last interpretation forms the basis of a probabilistic understanding of *C* involving the hitting region with random walk trajectories launched from the exterior of the particle^21^. Numerical methods utilizing this idea has been developed in the recent years^22,23^, and presently we can compute *C* with an accuracy easily better than 1% for particles of essentially any shape^16,22,23^.

Returning to our discussion of knots, there has been some previous interest in classifying knots in terms of energy concepts similar to *E*_C_. These definitions usually involve unphysical force laws or mathematical devices that insure that *E*_C_ remains finite for smooth curves (See discussion below). It is known that *C* for any smooth curve or any finite collection of smooth curves equals zero in three dimensions, so that *E*_C_ is then formally infinite^24^. This property evidently makes *E*_C_ unsuitable for discussing the topology of closed smooth curves^25^, but this limitation disappears as soon as the curve is endowed with a finite thickness or becomes fractal as in the case of the trajectories describing Brownian motion.

Hubbard and Douglas^15,26^ have recently shown that the translational friction coefficient *f*_t_ of Brownian particles having general shape is directly related to *C*,

3 $${f}_{{\rm{t}}}\approx 6\,\pi \,\eta \,C,$$

to a high degree of approximation, ${\mathscr{O}}(1\, \% )$. In Eq. (3), *η* is the fluid viscosity of the liquid where the particles are immersed, and the units of *C* are chosen so that the capacity of a sphere equals its radius. Eq. (3) has a simple physical interpretation; *f*_t_ describes the steady state diffusion of momentum away from diffusing object since *η* is the momentum diffusion constant.

The generalized Stokes–Einstein law, Eq. (3), provides a direct relation between knot shape, as measured by *C*, and the mobility *μ* of knotted DNA undergoing diffusion in solution,

4 $$\mu =1/{f}_{{\rm{t}}}\mathrm{.}$$

The sedimentation coefficients of a Brownian particle of general shape is proportional to *μ* ^27^. The general hydrodynamic–electrostatic relation, Eq. (3), is restricted to uncharged and rigid objects with a stick hydrodynamic boundary condition. Eq. (3) is based on the simple observation that angular averaging of the Oseen tensor gives rise to the Green’s function for the Laplacian^26^. Kholodenko and Rolfson employed an angular, and an additional configurational preaveraging approximation to relate the average knot crossing number 〈*m*〉 to an ensemble averaged “knot energy”^28^, and this work, in part, stimulated the present study. Our calculations of the knot energy using ZENO do not require a configurational preaveraging approximation, reducing the uncertainty in the analysis of the relation between *E*_C_ and 〈*m*〉.

In the present work, we argue about an approximate relationship between the Coulomb energy of a curve *E*_C_ and 〈*m*〉, thus giving a relation between chain mobility *μ* and 〈*m*〉. We first describe the coarse–grained molecular model used to generate polymeric knot configurations having different knot complexity and we use these configurations to test the aforementioned approximation, as well as to determine other shape descriptors that are related to knot complexity. We then explore the energy and shape properties of knotted rings having fixed length and different rigidity, as well as the properties of knotted rings having fixed rigidity and different length. We summarize our findings in the conclusion section.

## Knot Energies and Crossing Number

The Coulomb energy functional in Eq. (2) is a natural functional to define the complexity of knotted DNA since DNA is a highly charged macromolecule. It is well known that *E*_C_ is infinite for any smooth curve in 3 dimensions so this functional has not much been considered in relation to quantifying knot complexity. A generalized knot energy based on the potential |*R*|^−2^, the “Mobius energy”, has been much considered because this energy is invariant to reparametrization of the arc length, suggesting that this quantity might be a useful measure of knot complexity^28–30^. In this direction, Freedman *et al*.^30^ showed that the “regularized Mobius energy”,

5 $${E}_{{\rm{F}}}={\int }_{-n\mathrm{/2}}^{n\mathrm{/2}}\,d\tau {\int }_{\tau -n\mathrm{/2}}^{\tau +n\mathrm{/2}}d\tau ^{\prime} (|R(\tau )-R(\tau ^{\prime} {)|}^{-\beta }-|\tau -\tau ^{\prime} {|}^{-\beta }),$$

where *β* = 2, is directly related to the average crossing number of knotted curves,

6 $$\langle m\rangle +\mathrm{2/}\pi \,\le \,{E}_{{\rm{F}}}\mathrm{/2}\pi \mathrm{.}$$

Kholodenko and Rolfson^28^ considered an extension of the Mobius energy to the generalized potential |*R*|^−*β*^ and and achieved further simplification by ensemble averaging *E*_F_ over random walk paths. This simplification is associated with the fact that 〈*E*_C_〉 is finite for Brownian paths in 3 dimensions so that *E*_C_ divergence issues no longer exist when the paths defining the knotted structures are highly irregular^31,32,33^. Evidently, we need to consider the shape regularity (i.e., differentiability) of knotted curves in connection with the determination of 〈*E*_C_〉.

Based on the combined arguments of Freeman *et al*.^30^ and Kholodenko and Rolfson^28^, we conjecture that the Coulomb knot energy *E*_C_ should be nearly linear in the average crossing number 〈*m*〉,

7 $$\langle {E}_{{\rm{C}}}\rangle \sim \langle m\rangle ,$$

where the constant of proportionality in this scaling relation is unspecified. In the next sections, we explore the validity of this theoretically motivated approximation through a consideration of knotted polymer chains generated by molecular dynamics simulations and we use ZENO to determine 〈*E*_C_〉. Below, we find evidence supporting Eq. (7) for a selected family of knots of significance for the characterization of real DNA molecules and determine the prefactor in Eq. (7).

## Molecular Dynamics Simulations of Knotted Rings

### Generation of Semi–flexible Knotted Rings

**Figure 1:**
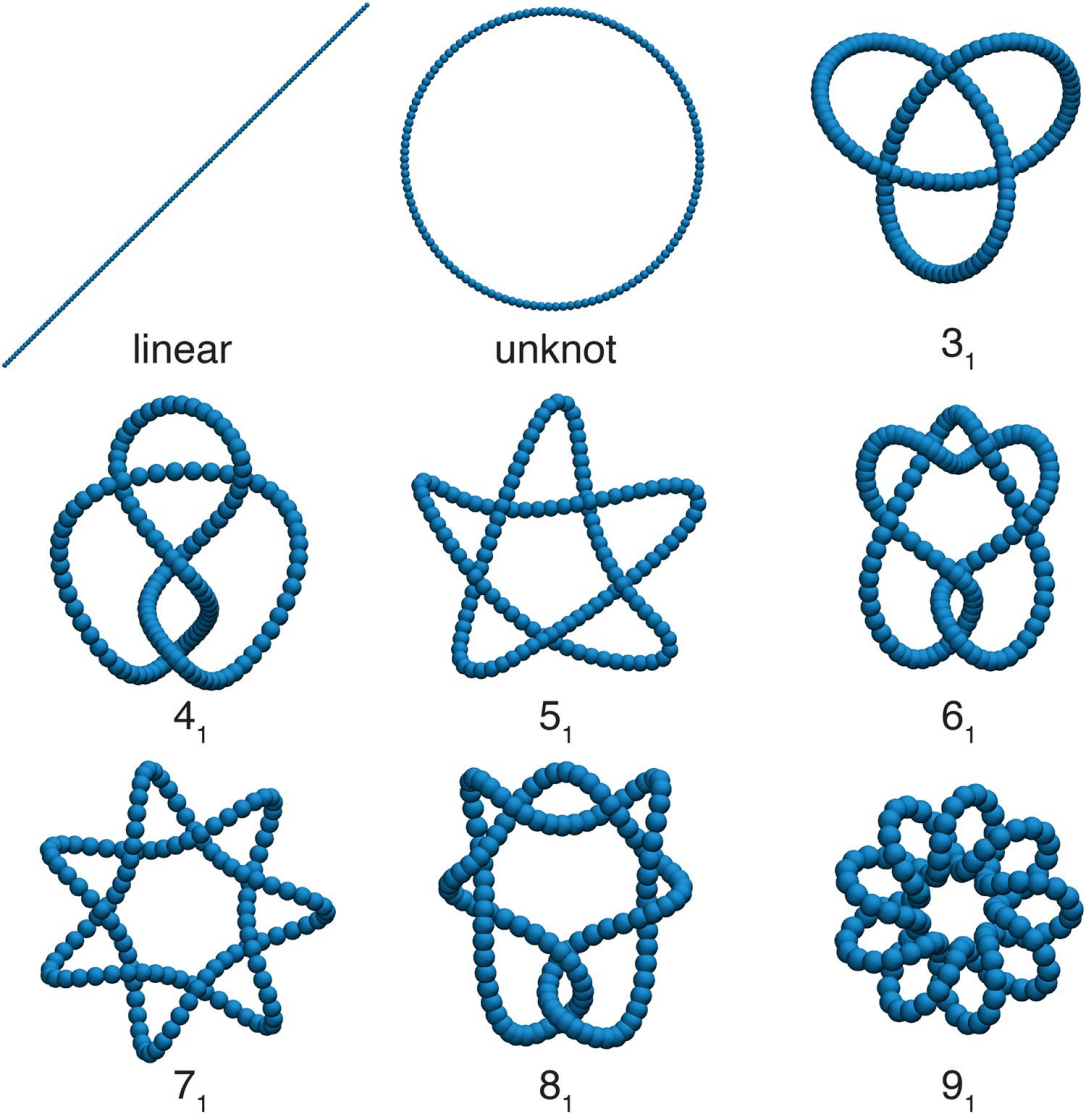
Initial configurations of the polymeric structures analyzed in this study. The properties of linear polymeric chains are used for comparison with those of knotted polymers. We follow the Alexander–Briggs knot classification notation^34^ where the main number is the minimal crossing number *m* and the subscript is an arbitrary number specifying sub–classes of knots having the same *m*. Knots having a subscript equal to unity are usually referred to “prime” knots and tend to be relatively “symmetric” in shape as class. All of the polymers on this figure are formed by 126 beads, corresponding to a length *L* = 352.8 nm, and a diameter *d* = 2.8 nm, appropriate for dsDNA^35^. The bead size has been scaled in the figure so that the whole polymer can be visualized on a common scale.

In this section, we describe a coarse–grained molecular model utilized in previously modeling of DNA in solution^36,37^ to generate the knotted polymeric configurations (Fig. 1). In this molecular model, each polymeric knot is represented by *L* = (63, 126, 200, or 252) connected beads (bead–spring model^38^). To generate the steric interaction among the beads, we use a Weeks–Chandler-Andersen (WCA) potential,

8 $${U}_{{\rm{WCA}}}(r)=\{\begin{array}{ll}4\varepsilon [{(\frac{\sigma }{r})}^{12}-{(\frac{\sigma }{r})}^{6}]+\varepsilon  & if\,r\le {2}^{\mathrm{1/6}}\,\sigma \\ 0 & if\,r > {2}^{\mathrm{1/6}}\,\sigma \mathrm{.}\end{array}$$

Here, *r* is the radial distance between the centers of two beads, and *σ* and *ε* are the length and energy Lennard–Jones parameters, respectively. Neighboring beads along the chain are connected via a finitely extensible, nonlinear elastic (FENE) anharmonic spring potential,

9 $${U}_{{\rm{FENE}}}(r)=-\frac{k{R}_{{\rm{0}}}^{2}}{2}\,\mathrm{ln}[1-{(\frac{r}{{R}_{{\rm{0}}}})}^{2}],$$

with the bond strength *k* = 30 *ε*/*σ* ^2^ and maximum bond extension *R*_0_ = 1.5 *σ*. In particular, we are interested in the properties of double–stranded DNA (dsDNA), we relate *σ* ≈ 2.8 nm to the diameter of dsDNA, a representative value for dsDNA in ≈1 mol l^−1^ of NaCl solution^35^, so that the knotted polymer lengths analyzed in this study corresponds to *L* = (176.4, 353.8, 560.0, 705.6) nm. We include a three–body bending potential *U*_b*end*_ among every three neighboring beads forming an angle *θ*,

10 $${U}_{{\rm{bend}}}(\theta )={k}_{{\rm{bend}}}[1-\,\cos (\theta )],$$

where *k*_bend_ is the bending constant and we consider *k*_bend_ = (1, 3, 5, 10, 20) *ε* to vary the knot polymeric stiffness. We characterize the rigidity of the polymer by computing the persistence length *l*_p_ for the linear polymeric chains, where *l*_p_ is defined as the average projection of the chain end–to–end distance **R**_e_ on the first bond of the chain **l**_1_^39^,

11 $${l}_{{\rm{p}}}=\langle {{\bf{R}}}_{{\bf{e}}}\cdot {{\bf{l}}}_{{\bf{1}}}\rangle /\langle {l}_{{\rm{1}}}\rangle \mathrm{.}$$

The values of *k*_b*end*_ indicated before lead to *l*_p_ = (5.8, 9.2, 13.7, 25.5, 50.2) nm for polymers having a linear topology.

Molecular dynamics (MD) simulations on this coarse–grained polymer model were performed to generate large ensembles of knot configurations. All simulations were performed at fixed number of particles, volume, and temperature (*NVT* ensemble). We chose temperature in the range 0.2 ≥ *T* ≥ = 3.0 *ε*/*k*_*B*_ and a Nosé–Hoover thermostat^40,41^ to generate and equilibrate chain ensembles. Here *k*_*B*_ is the Boltzmann constant. We first carry out our simulations for periods of time ≥ 10^7^ time steps *δt*, where *δt* = 0.006 *σ*(*m*/*ε*)^1/2^ to achieve the thermal equilibrium for each system. We performed our MD simulations by using the Large–scale Atomic Molecular Massively Parallel Simulator (LAMMPS)^42^. We report the average property for each system resulting from 4000 different configurations after it has reached thermal equilibrium. The property calculations were obtained by using the path–integration program ZENO^22,23,43^ based on a sampling of 10^6^ random walks. We use Visual Molecular Dynamics VMD^44^ to render representative configurations of the different knotted polymers.

### Generation of Canonical Knotted Rings

The characterization of knotted polymers is evidently complicated by the vast number of configurations that these polymers can have subject to topological constraints that define the knotted polymer type. This is a general problem in the recognition of a classes of objects sharing common topological or geometrical properties. A human, for example, normally has a head, two arms and two legs and articulation, points, or joints that allow a large number of possible configurations that humans explore in the course of their daily activities. The objective identification of humans, and other objects in data bases, has been facilitated by the identification of unique “canonical forms” associated with the entire class of objects that can serve to identify the object class^45–47^. In image recognition algorithms, canonical forms have been defined by associating an energy functional with a schematic representation of a member of the shape ensemble and the shape is then adjusted incrementally until the energy functional is extremized, subject to the geometrical invariants that define the class of objects.

**Figure 2:**
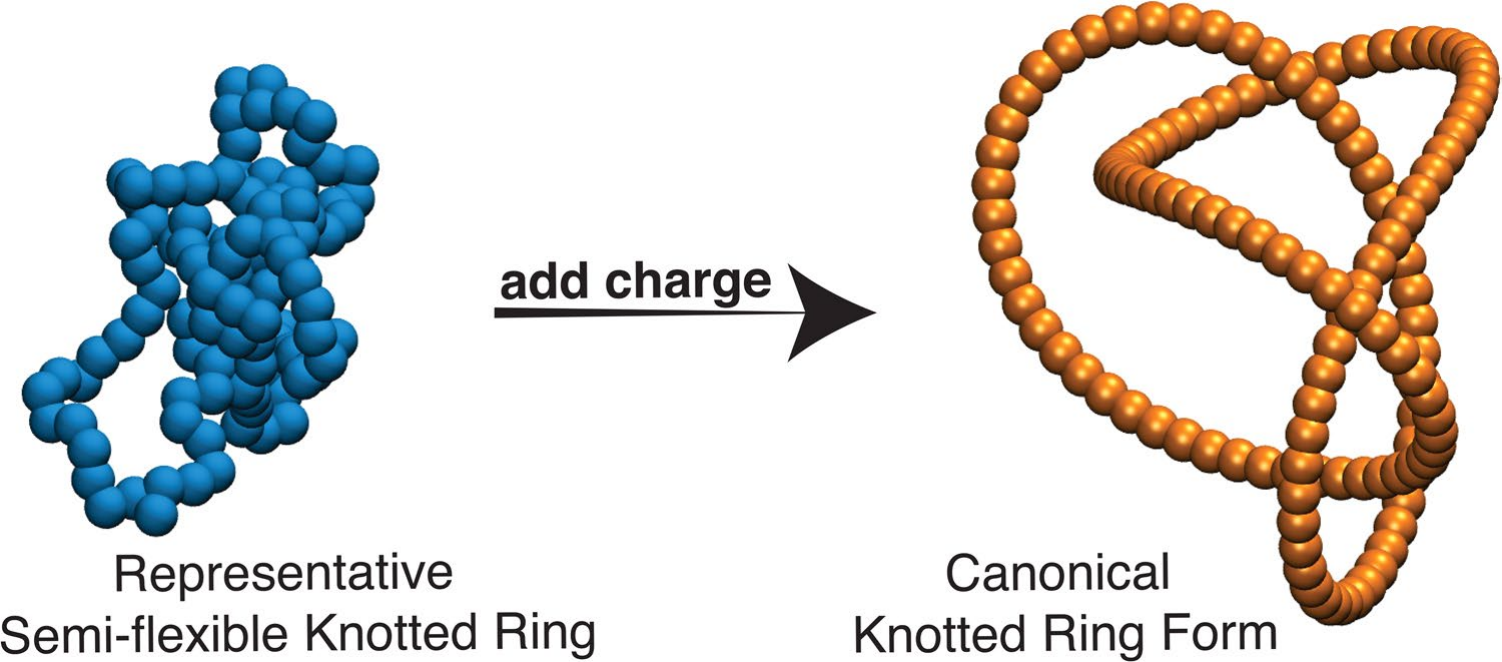
Representative configurations for a 4_1_ polymeric knot before (left) and after (right) including a repulsive charge interaction on the polymer beads. The addition of an electrostatic repulsive interaction reduces the average Coulomb energy and the fluctuations in the knotted ring shape arising from thermal fluctuations. The number of chain configurations decreases progressively with increasing *Z* until the knotted ring adopt an apparently unique configuration, the “canonical form”.

We follow this approach to classify knot types based on the Coulomb knot energy. In particular, we take any representative knot polymer configuration where the polymer chain is considered to be a conductor, and each bead has a fix charge and the beads are connected. We then allow the knotted rings to relax to the equilibrium configuration that minimizes the ensemble average Coulomb knot energy. By iterating this process and progressively increasing the charge magnitude on the beads of the polymer chain, we find the knotted polymers approaches an apparently unique “canonical” knot form for each class of knotted polymers. In particular, we achieve the generation of canonical knots by adding an electrostatic repulsive interaction *U*_coul_ among all the beads that form the polymer,

12 $${U}_{{\rm{coul}}}(r)=\{\begin{array}{ll}\frac{{Z}^{2}}{r} & \,if\,r\le \mathrm{4\ }\sigma \\ 0 & \,if\,r > \mathrm{4\ }\sigma ,\end{array}$$

where the charge *Z* is *Z* = 10. The qualitative idea of generating knots having minimal energy has been explored before before^25,48–50^ which are usually based on knot energies motivated by mathematical convenience rather than physical concerns. Figure 2 shows representative images for a 4_1_^34^ knot before and after introducing the repulsive charge interaction.

The application of this charging procedure reveals that a particular knot invariant as being of primarily significance in this classification scheme of knots, the minimal or essential crossing number *m*. The average crossing number 〈*m*〉 is obtained by averaging over the whole ensemble of possible knot configurations and this quantity is evidently larger than the minimal crossing number. In particular, 〈*m*〉 is larger for flexible chains than for rigid ones. Since *m* and 〈*m*〉 are important in our discussion below, we illustrate in greater detail how they are calculated.

**Figure 3:**
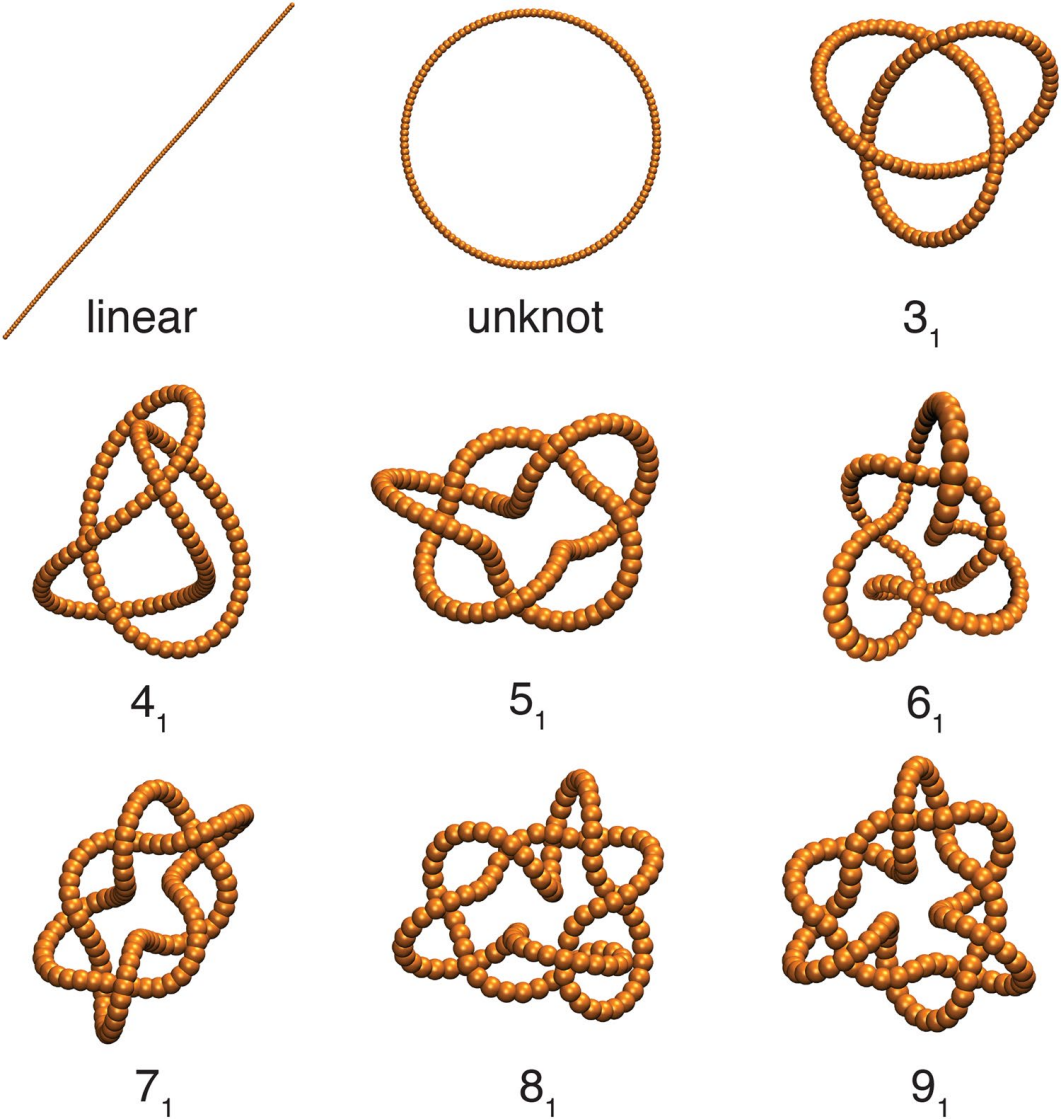
Representative rendering images for the ideal or “canonical” knotted polymeric structures generated by the process described in this section. All of the polymers on this figure are formed by 126 beads, corresponding to a length, *L* = 352.8 nm, and the diameter, *d* = 2.8 nm, appropriate for dsDNA in solution containing 1 mol l^−1^ NaCL^35^. The bead size has been scaled in the figure so that the whole polymer can be visualized on a common scale.

Figure 3 illustrates ideal canonical forms for knotted polymers having fixed *m*. It is apparent that the Coulomb canonical knots closely resemble “ideal” knots generated by increasing the polymer diameter incrementally rather than charging. In each case the knotted rings adapt an apparently unique “swollen” configuration^3^, although we are not aware of any rigorous proof of the uniqueness of this structure. Coulomb canonical knots provide a reference point in our discussion below of semi–flexible knotted polymers which exhibit highly complex and diverse configurations which are better characterized by 〈*m*〉 than *m*.

## Properties of Knotted Rings: Effect of Polymer Stiffness

In this section, we define the average crossing number 〈*m*〉 and explore its relation to 〈*E*_C_〉 and *m*. Additionally, we report the shape and size properties for knotted rings having fixed length *L* = 352.8 nm and fixed diameter *d* = 2.8 nm, and variable knot complexity and rigidity. The classification of the polymeric chains based on their rigidity is given by the calculation of their persistence length^39^. In our discussion below, we refer the persistence length *l*_p_ of linear polymeric chains having the same bending rigidity parameter *k*_b*end*_ as our measure of polymer rigidity.

### Average Crossing Number of Semi–Flexible Knotted Rings

**Figure 4:**
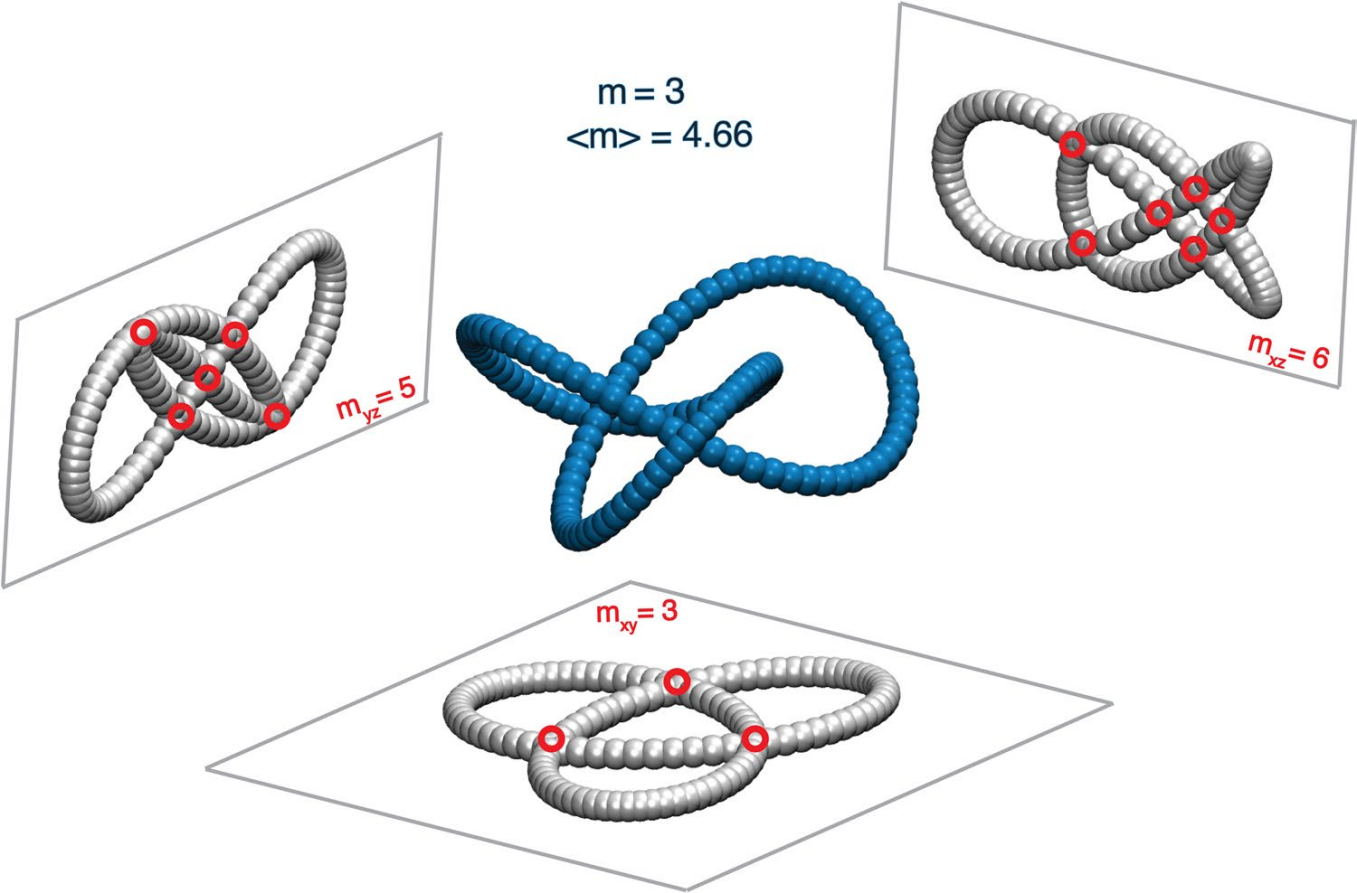
Example calculation for the average crossing number 〈*m*〉 for an initial configuration of 3_1_ knotted polymer. The knot is projected onto the *xy*, *xz*, and *yz* planes. We indicate the crossing points in each plane (i.e., 3 red circles in the *xy* plane) and we average over the crossing value determined in each plane 〈*m*〉. For this specific case, 〈*m*〉 = 4.66. For the flexible polymer cases, the averaging is performed over 4000 knotted polymer configurations generated as the knotted ring explores it configuration space.

We initially describe the methodology used to compute the average crossing number 〈*m*〉 for the thermally equilibrated knot configurations. For simplicity, we calculate 〈*m*〉 for a simple knot geometry. Figure 4 shows an example calculation of 〈*m*〉 for an initial configuration of a 3_1_ knotted polymer in the conveyed knot classification scheme^34^. Here, a knot having a crossing number *m* = 3 is projected onto the *xy*, *xz*, and *yz* planes. We count the crossing points in each plane, giving by the intersection of the monomers (e.g., 3 red circles shown on projected polymer in the *xy* plane) and then we average over the crossing value determined in each plane to determine 〈*m*〉. The calculation of 〈*m*〉 for an individual knotted polymer involves projecting the polymer onto an infinite number of planes having a relative angular distribution having uniform distribution. This type of avergaing is simplified by our Monte Carlo Sampling procedure which explores all angular orientations of the knotted polymer while the planes remain fixed. Having three orthogonal planes improves the sampling, but is not required in our method of angular averaging through our molecular dynamics based exploration of polymer conformational space. For the thermal equilibrated knotted polymers, 〈*m*〉 is obtained by averaging the 4000 configurations. Figure 4 shows 〈*m*〉 as a function of *m* for knotted polymers having different stiffness and fixed length *L* = 352.8 nm.

**Figure 5:**
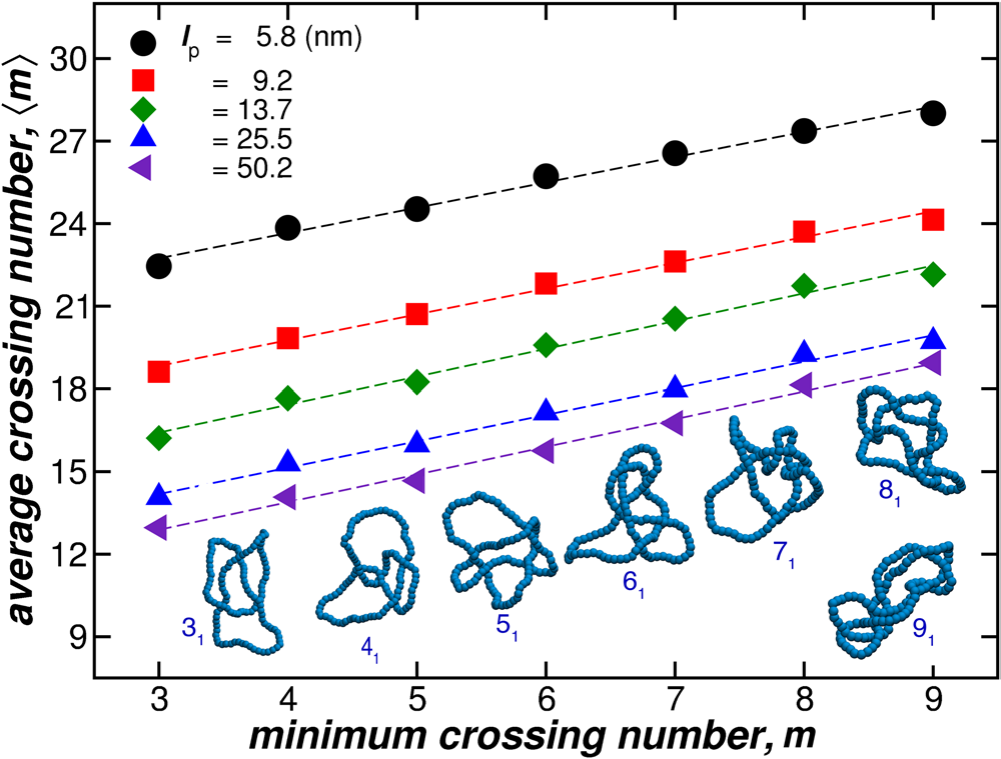
The average crossing number 〈*m*〉 as a function of the minimum crossing number *m* for polymeric knots having different stiffness (See legend) and fixed length *L* = 352.8 nm. The images corresponds to representative knot configurations for the polymers interacting with a bending energy amplitude *k*_bend_ = 10 *ε*, corresponding to linear chains having *l*_p_ = 25.5 nm (blue triangles).

For flexible knot polymers, it is more challenging to visualize its minimal crossing number *m*, but using the procedure described above, we can compute 〈*m*〉. Figure 5 shows 〈*m*〉 as a function *m* for knotted polymers having different stiffness, but a fixed length, *L* = 352.8 nm. These images correspond to representative knot configurations for the polymers interacting with a bending energy amplitude *k*_b*end*_ = 10 *ε* (blue triangles). We find an approximately proportional relation between 〈*m*〉 and *m* where increasing polymer stiffness shifts the curves downwards, reflecting the fact that rigid polymers have a smaller average number of crossing points.

### Relations between 〈*E*_C_〉, *m*, and 〈*m*〉

**Figure 6:**
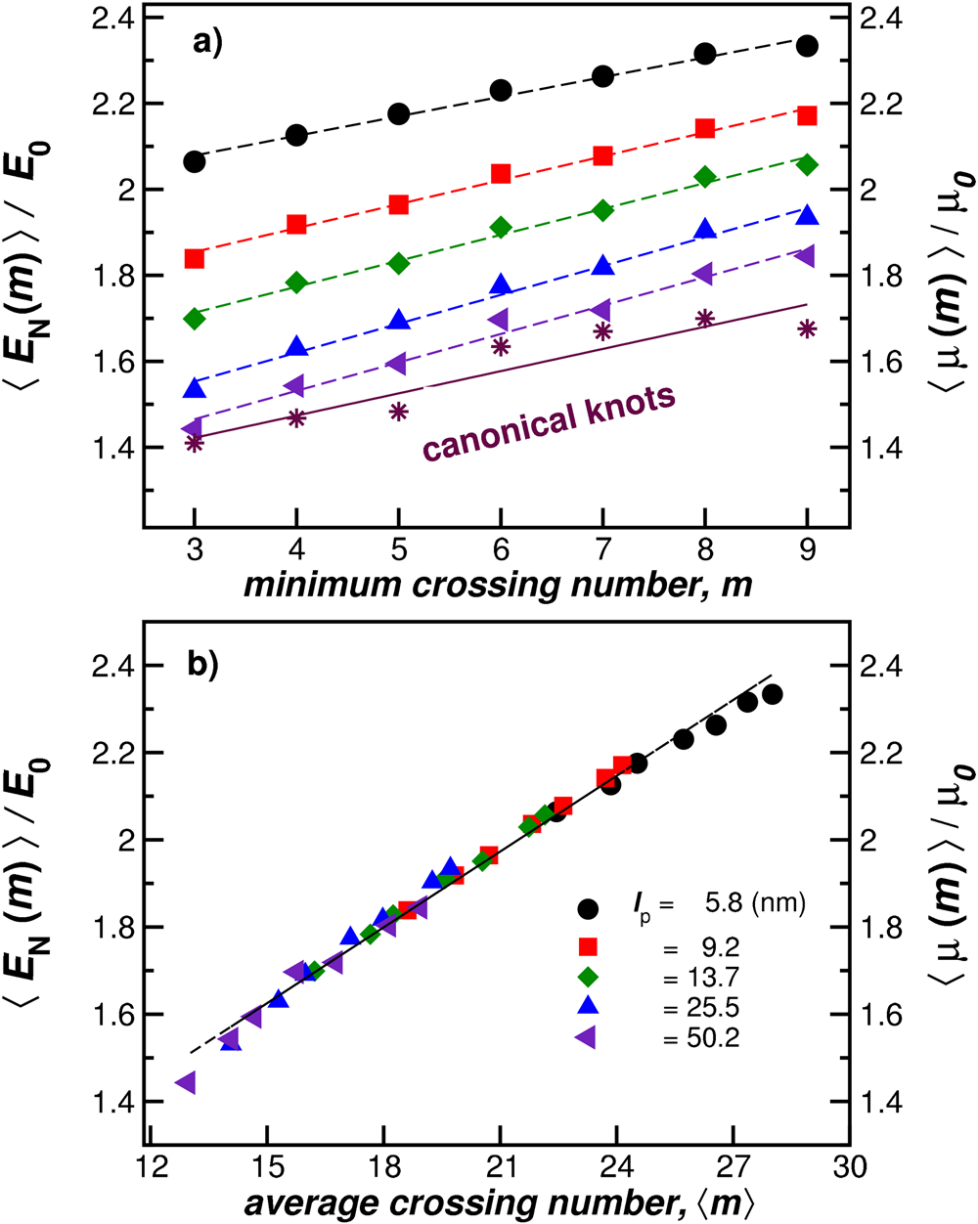
The average Coulomb energy 〈*E*_C_〉 as a function of the minimum crossing number *m* (upper panel) and average crossing number 〈*m*〉 (lower panel) for polymers having the same length *L* = 352.8 nm and different chain rigidities. Here, 〈*E*_C_〉 has been normalized by the energy of the canonical form of the unknotted polymer, *E*_0_, which corresponds to the lowest energy configuration for a polymer of fixed length, diameter, and rigidity. We find a linear relationship between 〈*E*_C_〉 and *m*. However, when we plot 〈*E*_C_〉/*E*_0_ as a function of 〈*m*〉, all the data collapse onto a universal curve (lower panel).

We next compute the average Coulomb energy 〈*E*_C_〉, *m*, and 〈*m*〉 for the polymeric knots configurations generated using the coarse–grained model described in the previous section for a selected family of knot types relevant to the characterization of real DNA. Figure 6 shows the average Coulomb energy 〈*E*_C_〉 as a function of the minimum crossing number *m* (a) and average crossing number 〈*m*〉 (b) for polymers having the same length *L* = 352.8 nm and different degree of stiffness. All our data has been normalized by the Coulomb energy for the canonical unknotted polymer, *E*_0_, which corresponds to the lowest energy structure among all knotted rings. We find a linear relationship between 〈*E*_C_〉 and *m*, where the intercept evidently depends on the rigidity of the polymer chain. However, 〈*E*_C_〉 normalized by *E*_0_, is nearly a universal function of 〈*m*〉, as its indicated in Fig. 6(b). We also see from Fig. 6(b) that the average mobility 〈*μ*(*m*)〉 of knotted polymers having a fixed *m* is directly proportional to the Coulomb knot energy to within the good approximation; See Eq. (4). The mobilities of knotted polymers in sedimentation measurements have been observed to exhibit the same linear scaling between 〈*μ*(*m*)〉 and 〈*m*〉^49^. Recent sedimentation measurements on knotted polymers having ideal knot configurations (defined by strong repulsive excluded volume interaction rather than charge interaction) also follow this scaling relation to a reasonably good approximation, although the data is somewhat noisy. Figure 6(b) confirms the proposed relation between 〈*E*_C_〉 and 〈*m*〉 in Eq. (7). We have not made an exhaustive sampling of all knot types in our analysis here, but rather have focused on knots that seen to be of practical significance in relation to observations on real DNA molecules. Further work shall include other less “symmetric” knots to fully verified the generality of the linear relation between 〈*m*〉 and 〈*E*_C_〉.

### Basic Measurement of Size and Shape of Knotted Polymers Relevant to Experimental Characterization

To characterize the shape of polymers and particles, it is common to determine the radius of gyration tensor, ***R***_**g**_, which can be experimentally obtained by scattering techniques and it is formed by 9 components,

13 $${{\boldsymbol{R}}}_{{\bf{g}}}^{{\bf{2}}}=(\begin{array}{ccc}{R}_{{{\rm{g}}}_{{\rm{xx}}}}^{2} & {R}_{{{\rm{g}}}_{{\rm{xy}}}}^{2} & {R}_{{{\rm{g}}}_{{\rm{xz}}}}^{2}\\ {R}_{{{\rm{g}}}_{{\rm{yx}}}}^{2} & {R}_{{{\rm{g}}}_{{\rm{yy}}}}^{2} & {R}_{{{\rm{g}}}_{{\rm{yz}}}}^{2}\\ {R}_{{{\rm{g}}}_{{\rm{zx}}}}^{2} & {R}_{{{\rm{g}}}_{{\rm{zy}}}}^{2} & {R}_{{{\rm{g}}}_{{\rm{zz}}}}^{2}\end{array})\mathrm{.}$$

**Figure 7:**
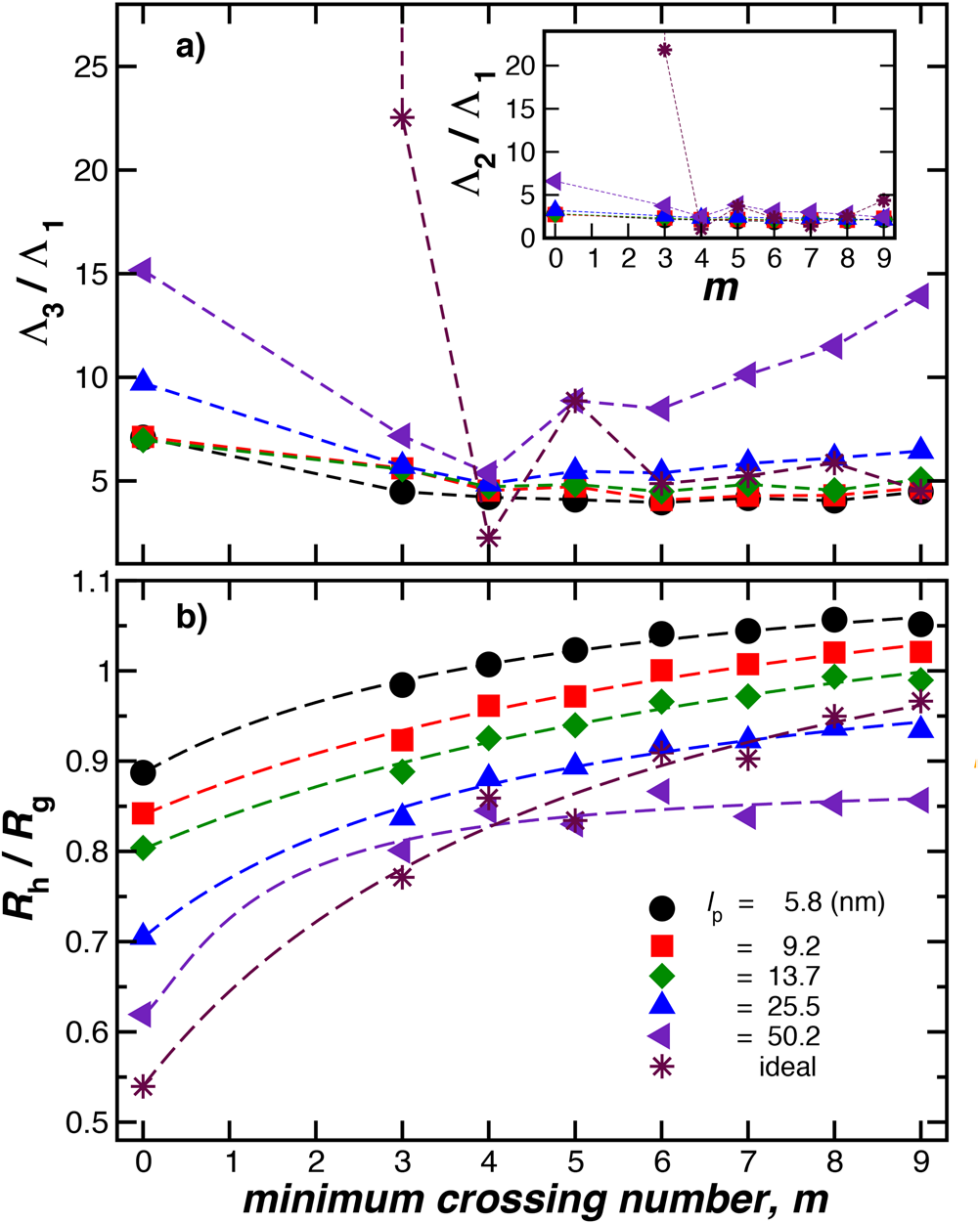
Shape characterization of the knotted ring polymers as a function of the minimum crossing number *m*. Here, the polymeric knots have fixed length *L* = 352.8 nm. In the upper panel, the ratio between the largest Λ_3_ and the smallest Λ_1_ eigenvalues of the radius of gyration tensor. The inset shows Λ_2_/Λ_1_ to complete the shape description of the knots as an object embedded in a spheroid whose main axis are Λ_1_, Λ_2_, Λ_3_. Higher ratios indicate higher anisotropy of the polymers. In the lower panel, we show the ratio *R*_h_/*R*_g_, where *R*_h_ is simply equal to *C*. This ratio indicates the changes between and open structure (small values) to a more closed compact one (higher values).

${R}_{{\rm{g}}}^{2}$ is the norm of the tensor and it can be determined from the tensor trace,

14 $${R}_{{\rm{g}}}^{2}=({{\rm{\Lambda }}}_{1}+{{\rm{\Lambda }}}_{2}+{{\rm{\Lambda }}}_{3})\mathrm{/3,}$$

where Λ_i_ are the eigenvalues of ${{\boldsymbol{R}}}_{{\bf{g}}}^{{\bf{2}}}$ and Λ_1_ ≤ Λ_2_ ≤ Λ_3_. The ratios Λ_3_/Λ_1_ and Λ_2_/Λ_1_ constitute shape descriptors and represent each knot as an ellipsoid which main axis are Λ_1_, Λ_2_, Λ_3_ and also define the average anisotropy of the particle or polymer^52^. The ratio *C*/*R*_g_ is another important shape descriptor, which indicates the changes between and open structure (small values) to a more closed compact one (higher values)^53^. For instances, *C*/*R*_g_ = 0 approaches 0 for a needle and this ratio for a solid spherical particle, *C*/*R*_g_ = 1.29^54^. Figure 7 shows these shape descriptors for knotted polymers having fixed length (*L* = 352.8 nm) and different polymer rigidities.

**Figure 8:**
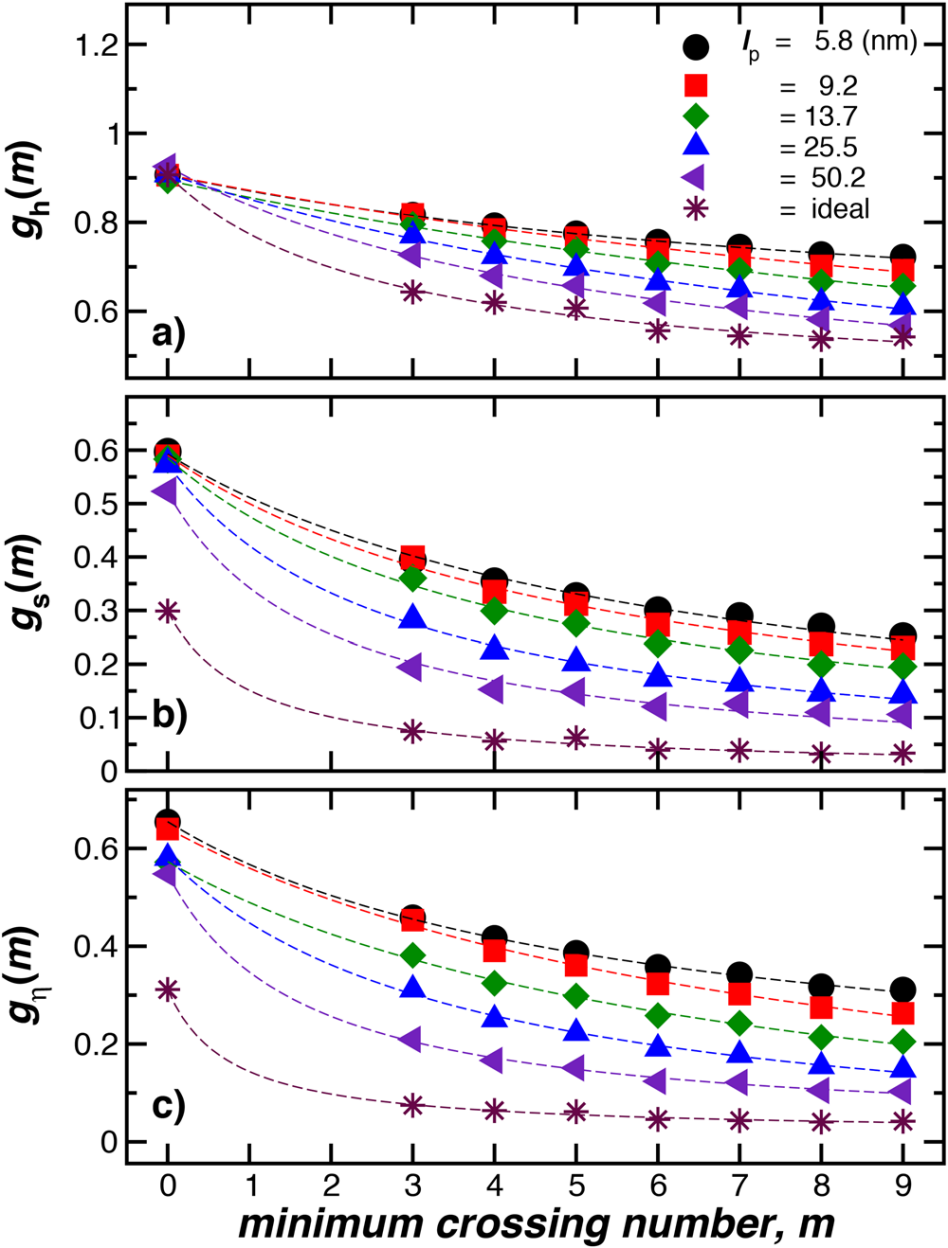
Basic solution characterization of knotted rings having variable rigidity. We compare size related properties of knotted and linear polymers having the same length, *L* = 352.8 nm. The different colors and symbols represent polymers having different stiffness specified by the persistence length of the linear polymer, *l*_p_, indicated in the inset. In panel (a), hydrodynamic radius ratios *g*_h_(*m*), in panel (b), radius of gyration ratios *g*_s_(*m*), and in panel (c), intrinsic viscosity ratios, *g*_*η*_(*m*). These ratios evidently decrease with increasing *m* and chain stiffness.

We next consider basic measures that are commonly used to determine the topological structure of macromolecules^21^. In particular, we directly compare the knotted polymer properties to those of a linear polymer having the same molecular mass,

15 $${g}_{{\rm{h}}}(m)\equiv {R}_{{\rm{h}}}(m)/{R}_{{\rm{h}}}({\rm{lin}}),$$

16 $${g}_{{\rm{s}}}(m)\equiv {R}_{{\rm{g}}}^{2}(m)/{R}_{{\rm{g}}}^{2}({\rm{lin}}),$$

17 $${g}_{\eta }(m)\equiv [\eta (m)]/[\eta ({\rm{lin}})],$$

where *C* ≈ *R*_h_ and [*η*] is the intrinsic viscosity of the polymeric structure. [*η*] is proportional to the average electric polarizability tensor and describes how the addition of the polymer alters the viscosity of the polymer solution in the low polymer concentration limit^14^. We plot these transport property ratios as a function of the minimum crossing number *m* in Fig. 8. We see that these ratios generally decrease with increasing *m* and increasing chain stiffness.

**Figure 9:**
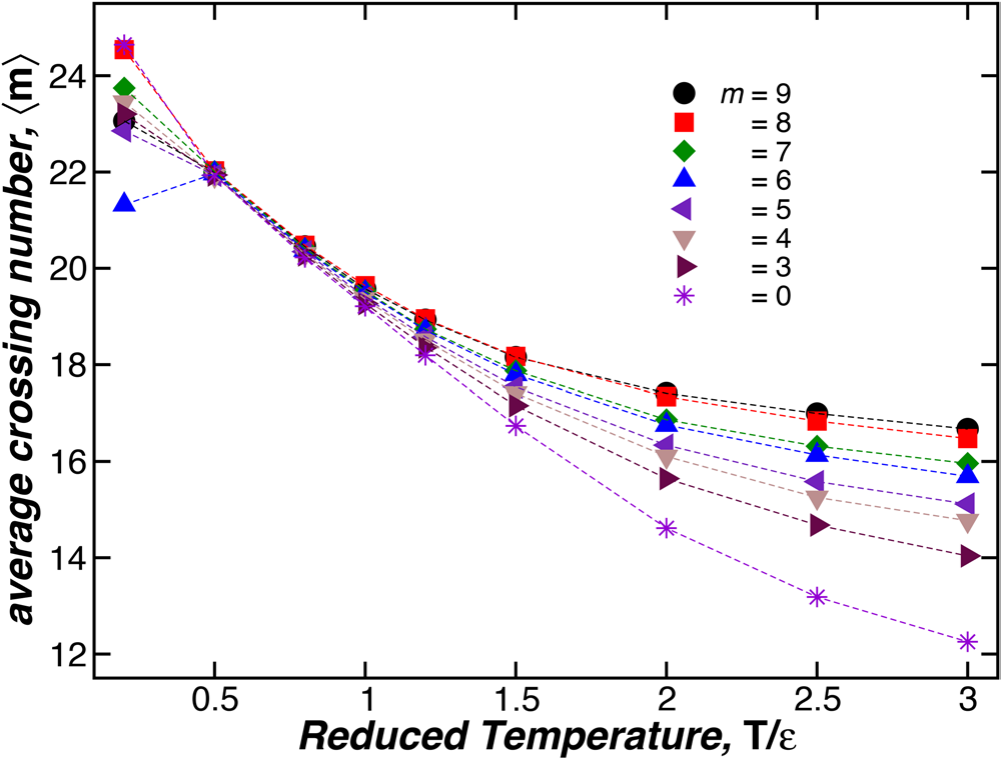
Average crossing number 〈*m*〉 as a function of the reduced temperature *T*/*ε* for polymeric knots having fixed chain stiffness *l*_p_ = 5.8 nm, diameter *d* = 2.8 nm, and length *L* = 176.4 nm. Dashed lines are a guide to the eye.

Comparison between our *g*_h_, *g*_s_, and *g*_*η*_ calculations with experiments on synthetic polymers is complicated by the fact that the knot complexity of ring polymers is normally “locked in” at the time of synthesis, leading to structures that are topologically polydisperse, meaning that many different types of knotted polymers can be generated in the synthesis process. Specifically, the end–linking of the linear polymer chain precursor molecules is often performed in a poor solvent where there are appreciable self–attractive polymer–polymer interactions in order to increase the probability of the chain ends to react and to form a ring. While these thermodynamic conditions do enhance ring formation, they can also be expected to greatly influence the probability of the rings to be knotted. This general trend can be appreciated by considering how 〈*m*〉 varies with *T* for rings having a fixed *m*. Figure 9 shows 〈*m*〉 as a function of the reduced temperature *T*/*ε*, where *ε* is the well depth parameter of the Lennard–Jones interaction potential. We see that 〈*m*〉 varies from a large value at low temperature where the knotted rings are in a collapsed configuration towards a value that gradually seems to be approaching the minimal crossing number *m* with increasing *T*. Unexpectedly, 〈*m*〉 becomes insensitive to *m* for a specific value of *T*. This variation of 〈*m*〉 with *T* is remarkably similar to the variation of the number of nearest–neighbor contacts of self–avoiding walks with an attractive nearest–neighbor interaction^55,56^, which is natural since the projected structure of the knotted polymers on a planar surface (See Fig. 4) has the form of a branched polymeric structure.

This “memory effect” of the knot complexity of ring polymers in solutions on the thermodynamic conditions of cross–linking means that the *g*–ratios are defined as a weighted average,

18 $$g=\sum _{m=0}^{{m}_{{\rm{\max }}}}\sum _{i\mathrm{=0}}^{{i}_{{\rm{\max }}}}\,{g}_{i}(m)P(i,m),$$

where *P*(*i*, *m*) is the probability of the ring polymer is in a topological state having a minimal cross–linking number *m* and subclass *i*. For polymeric rings of moderate length *L* that is synthesized in a good solvent, we can expect almost all the rings to be in the unknotted state (*m* = 0) so that *g* ≈ *g*_0_(*m* = 0), but knots of increasing complexity should arise if the synthesis is performed under poor solvent conditions. The *g*–ratios for molecules synthesized in this way are inherently *non–universal*, even in the limit, *L* → ∞. This type of memory effect also arises in the cross–linking of macroscopic polymer networks^57^ and individual polymers^58^.

The fact that the knot complexity depends on chain length for even self–repelling chains adds to the variation of these *g*–ratios. Future work will evidently need to focus on the dependence of *P*(*i*, *m*) on solvent quality and chain flexibility to enable the computation of *g*–ratios for quantitative comparisons of our calculations to experiment.

## Properties of Knotted Rings: Effect of Polymer Length

The calculations above were made for knotted polymers having a fixed length and diameter. Short chains are inherently “stiffer” so that we might expect that increasing the chain length should have an effect size similar to increasing the chain rigidity. In this section, we confirm this expectation through direct computation of the properties of knotted rings having different lengths and chain stiffness, *l*_p_ = 50.2 nm, and chain diameter *d* = 2.8 nm. This choice of chain parameters is appropriate to describe double–stranded DNA in solution at salt concentrations sufficiently high for charge interaction to be screened (1 M NaCL).

### Dependence of 〈*E*_C_〉 and 〈*m*〉 on Chain Length

**Figure 10:**
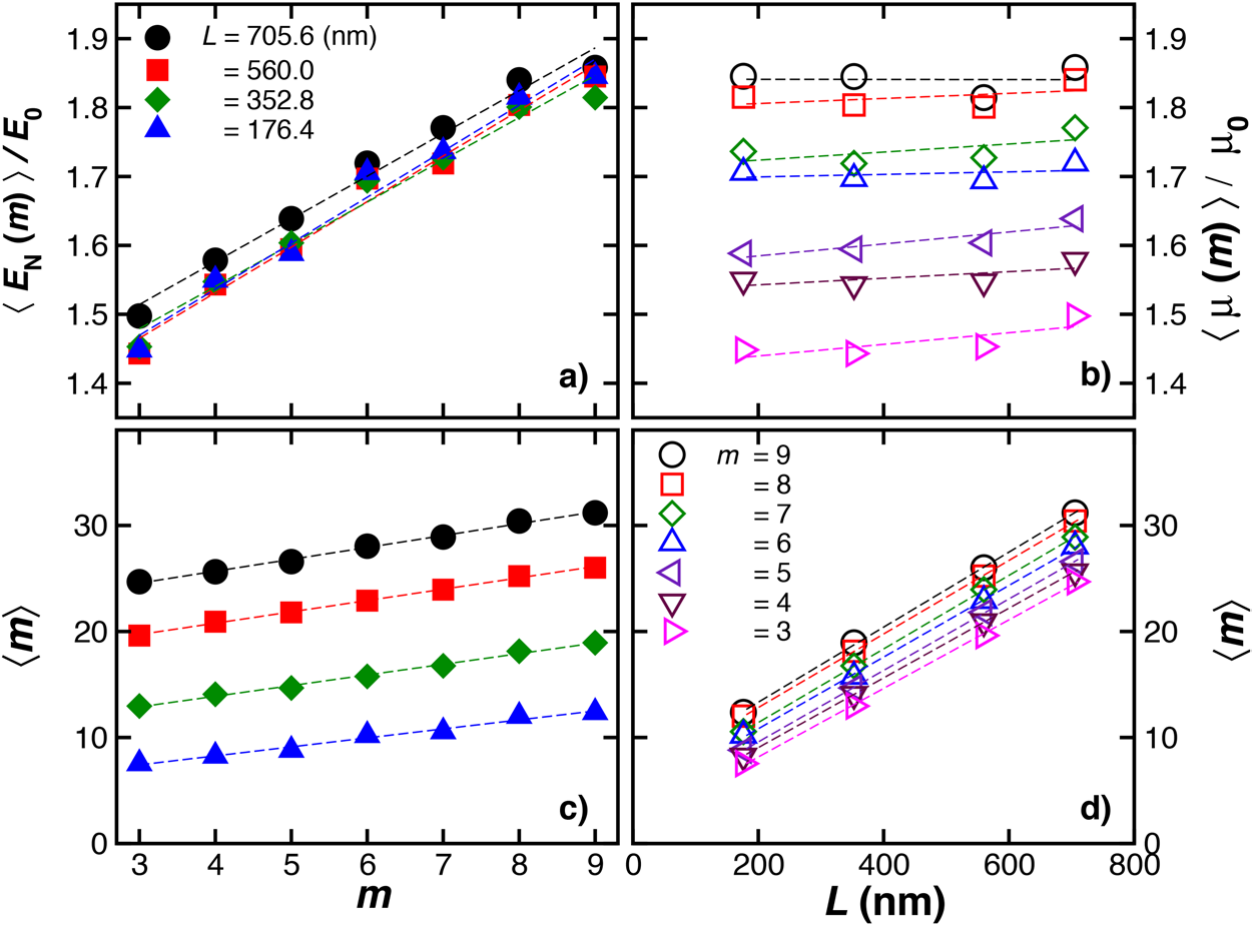
Knot energy, mobility, and average crossing number. The average Coulomb energy 〈*E*_C_〉 normalized by the canonical form energy *E*_0_ of unknotted polymer for variable *L* and fixed chain rigidity (*l*_p_ = 50.2 nm) and diameter (*d* = 2.8 nm). Relative mobility, 〈*μ*_N_〉/*μ*_0_. (**c**) Average crossing number 〈*m*〉 as a function of (**c**) the minimum crossing number *m* and (**d**) the knotted polymer length *L*.

Figure 10(a) shows the reduced energy 〈*E*_C_(*m*)〉/*E*_0_ and relative mobility 〈*μ*(*m*)〉/*μ*_0_ as a function of *m* (panel a) and length *L* (panel b). We find that 〈*E*_C_(*m*)〉/*E*_0_ increases linearly with *m* and varies weakly with *L*. On the other hand, 〈*m*〉 varies nearly linearly on *m* and *L*, as illustrated in Fig. 10(c,d), respectively. Larger chains exhibit a larger number of average crossing points 〈*m*〉 for all *m*. Correspondingly, Fig. 5 shows that more flexible chains having a fixed length have a greater average number of crossing points.

### Size and Shape of Knotted Polymers Having Different Chain Length

**Figure 11:**
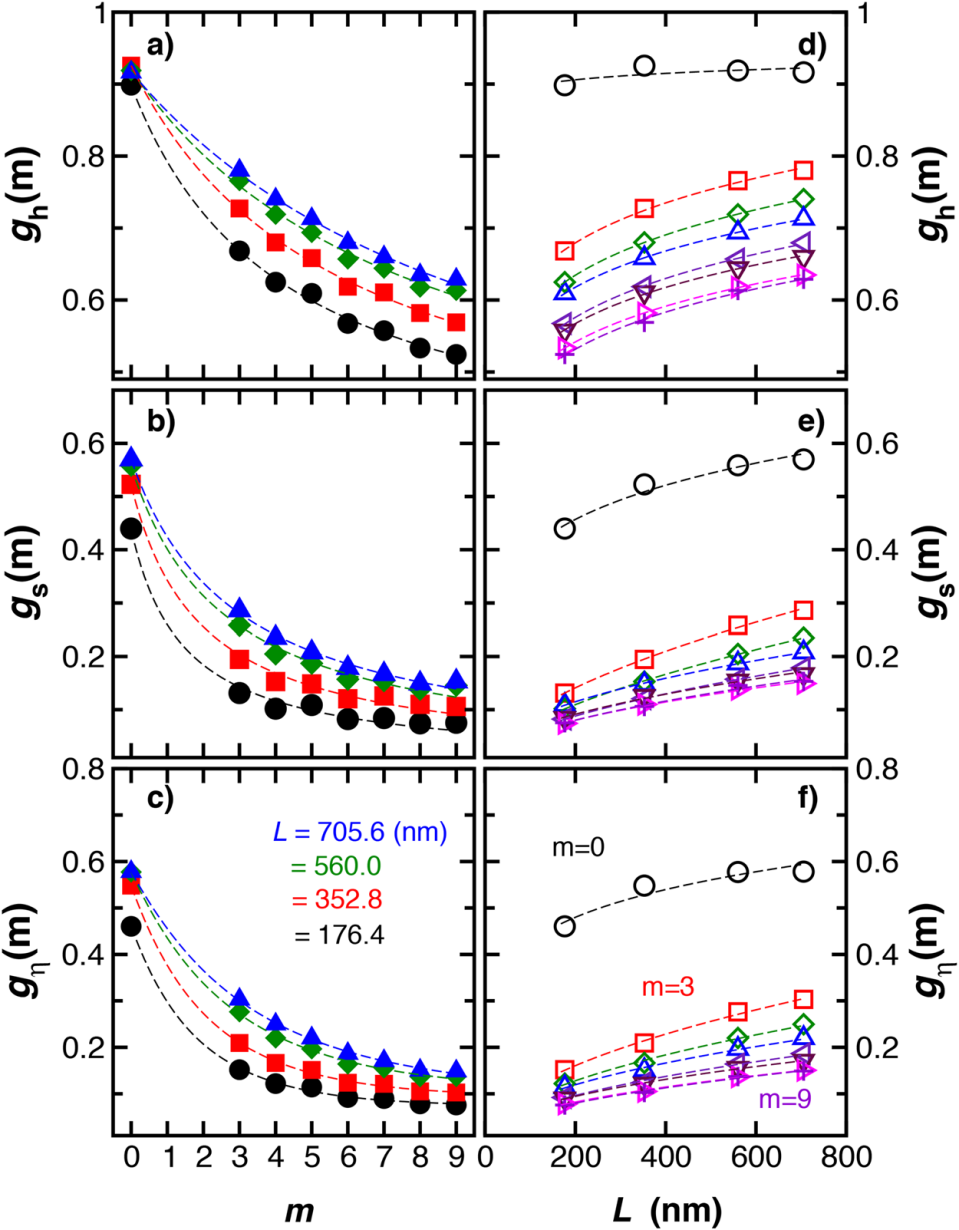
The knot–linear polymeric ratios as a function of *m* polymer length *L* for chains having fixed chain rigidity (*l*_p_ = 50.2 nm) and diameter (*d* = 2.8 nm). The ratios decrease with knot complexity and increase with the knotted ring length.

The size ratios, *g*_h_(*m*), *g*_s_(*m*), and *g*_*η*_(*m*) also depend on the chain length; these ratios being larger for larger chains in the case of polymers having a stiffness and diameter compatible with double–stranded DNA, as it is illustrated on Fig. 11. These basic size ratios become progressively smaller with increasing *m*, a trend similar to star branched polymers having an increasing number of arms^59^. Again, we note that this is a natural trend since the projection of a knotted polymer onto a plane is a branched polymer and the *g*_i_ ratios for branched polymers tend to decrease with the degree of branching^59^.

The chain length range in our study is rather limited and our uncertainties in estimating asymptotic power and law scaling of size measurements (*R*_h_, *R*_g_, and [*η*]) and correspondingly dimensionless ratios *g*_h_(*m*), *g*_s_(*m*), and *g*_*η*_(*m*) in the long chain limit are probably large. Previous studies have compared the ZENO model estimates of *R*_h_, *R*_g_, and [*η*] for linear chain dsDNA over a very large range of mass range where quantitative agreement of the modeling with a worm–like chain model with an appropriate diameter and persistence length was found^60^. The scaling properties of knotted polymers relating to size in the long chain limit were also investigated extensively in a previous computational study^61,62^. However, this former work did not emphasize the ratios *g*_h_(*m*), *g*_s_(*m*), and *g*_*η*_(*m*) and the importance of the knot complexity *m* on the properties of knotted polymers having fixed length or the influence of chain rigidity on the properties of knotted polymers.

**Figure 12:**
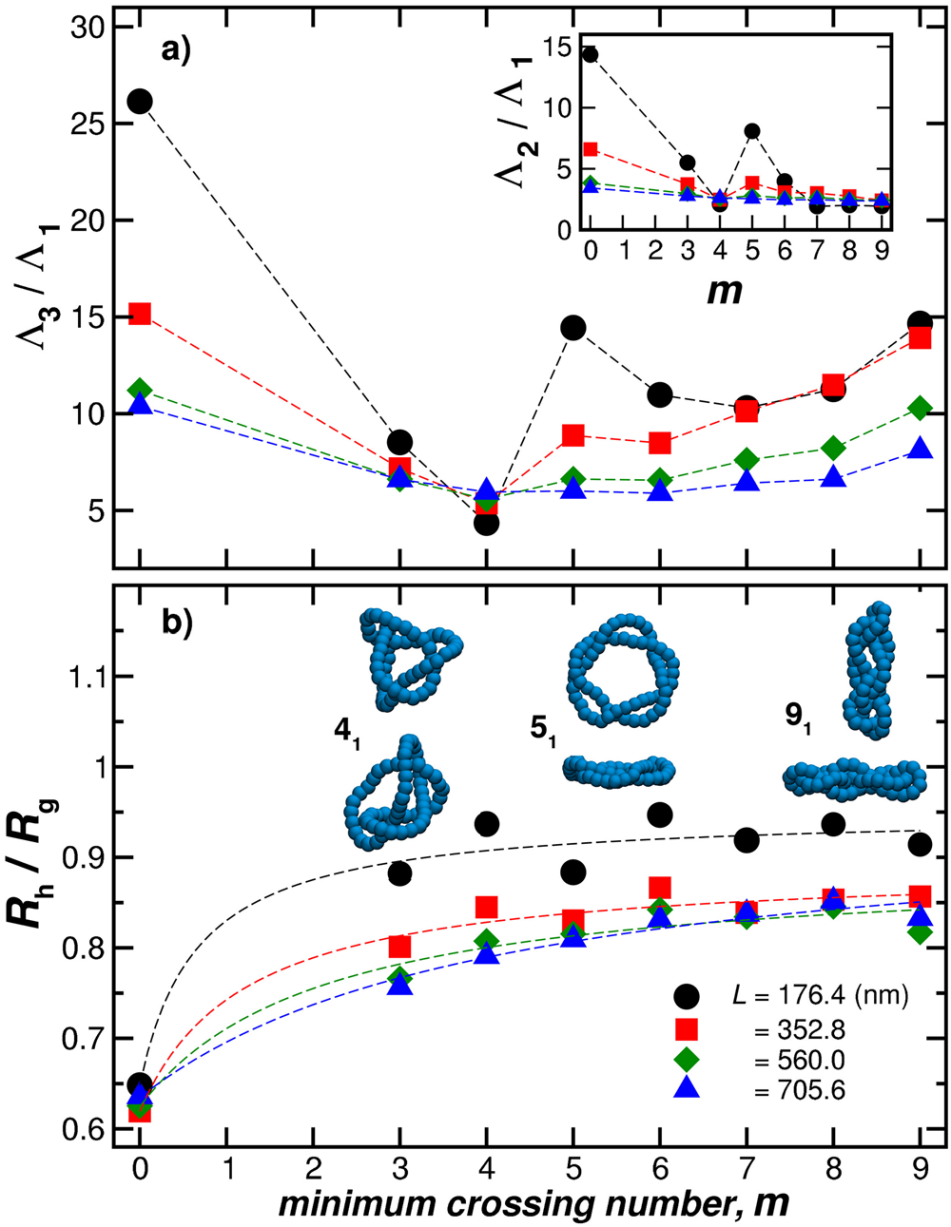
Shape characterization of the knotted ring polymers as a function of the minimum crossing number *m* for polymeric knots having fixed rigidity *l*_p_ = 50.2 nm and diameter *d* = 2.8 nm. In the upper panel, the ratio between the largest Λ_3_ and the smallest Λ_1_ eigenvalues of the radius of gyration tensor. The inset in panel (a) shows the ratios Λ_2_/Λ_1_ which provide a basic measure of the anisotropy of the shape of the knotted polymer. In (b), *R*_h_/*R*_g_ gets smaller with increasing length and approaches a saturation value with increasing knot complexity. The images in panel (b) are representative configurations taken from different orientations to show the anisotropy for knotted polymers having *L* = 176.4 nm.

Figure 12(a), and its inset, shows the eigenvalue ratios, Λ_3_/Λ_1_ and Λ_2_/Λ_1_, respectively, of the radius of gyration tensor as a function of the minimum crossing number *m* for polymeric knots having fixed rigidity *l*_p_ = 50.2 nm and diameter *d* = 2.8 nm. The variation of the average shape of knotted polymers with chain length is rather complex, exhibiting a relatively sharp change near *m* = 4, similar to star polymers having 5 to 6 arms^63^. With increasing crossing number *m*, the knotted polymer becomes more spherical, when the polymer is stiff. An examination of the resulting knotted stiff polymers for *m* > 4 indicates that these structures are more like woven sheets than a sphere, a phenomenon that we did not expect.

## Conclusions

Some important conclusions can now be drawn from the above relations. First, we have confirmed that the average Coulomb energy 〈*E*_C_〉 provides a natural measure of knot complexity that is directly related to measurements of DNA and often knotted structures. Simple knotted chains should have a lower average knot energy *E*_C_ and the “unknot” (*m* = 0) should have the lowest or “ground state” knot energy (conjuncture). From the discussion above, the average crossing number 〈*m*〉 is a related measure of knot complexity and indeed these functionals are proportional to a good approximation, Eq. (7), for the knots considered in this studied. Knots with higher complexity and less symmetry will be tested in a follow up study. The hierarchy of knot complexities, as reflected by the energy 〈*E*_C_〉, is directly reflected in the translational mobilities of knotted polymers through the generalized Stokes law, Eq. (3). The Coulomb energy thus directly pertains to an understanding of the mobility of knotted DNA. We emphasize that the proportionality relation between 〈*E*_C_〉 and *m* does not provide an obvious explanation of the proportionality between the electrophoretic migration speed and 〈*m*〉. While Stokes law is clearly appropriate for dilute polymer solutions the factors that governs chain mobility in gels are still not completely understood^64,65^.

We also draw on ideas introduced in the field of image recognition to define “ideal knots” characterizing distinct families of knots sharing a knot complexity defined by the crossing number *m*, along with other indices prescribed by convention. In particular, we define canonical knots as polymer configurations that results from charging the beads of our knotted polymer. Increasing this charge progressively, and then letting the system relax after each step, leads to an apparently unique knotted ring conformation that maximizes *C* and minimizes the Coulomb energy. In the imaging processing context, the energy function is normally taken to be a more complex than the Coulomb energy, while this energy functional is quite natural for DNA, a highly charged polyelectrolyte in the absence of much salt added to solution. These “canonical knots”, and their properties provide a natural point of comparison to rings of variable flexibility and no charge where the conformations have numerous complex forms with statistically define average properties that define their properties. We note that there has been a previous definition of ideal knots by progressively increasing the size of the beads in the chain where excluded volume repulsion is enforced^3^. Their conformations appear to be rather geometrically similar to our canonical form knots, but we prefer our definition since it has a more physical motivation in relation to DNA.

One of the other problems arising in characterizing knotted ring polymers, and predicting the properties that derive from such topologically defined polymers, is that it is often difficult to control the knot complexity in their synthesis. Nature has evolved enzymes to regulate chain topology in DNA, but synthetic chemists have a much more limited control of knot complexity, e.g., controlling the solvent quality conditions under which the chains are linked together. Under such conditions, it is imperative to have measurement methods and validated theoretical models that quantify how knot complexity influences average molecular shape along with standard measurements of hydrodynamic properties (*R*_h_, [*η*], *S*) and static (*R*_g_) size that are normally used to characterize polymers in solution^27^. We calculate all these basic polymer solution characterization properties as a function of the knot complexity *m* chain stiffness *l*_p_ and over a range of chain length, allowing an estimation of long chain limit values of *g*_h_, *g*_*η*_, and *g*_s_. The range of knot complexities explored is not exhaustive, but rather representative of the classes of knots found in the characterization of real DNA and presumably real synthetic macromolecules. We expect these results to be of great use in characterizing knotted polymers in solution.

We emphasize that the analytic calculation of the hydrodynamic properties of even flexible linear polymers, beyond a mean–field theory approximation, has long eluded theoretical description. The errors in existing theories in case of flexible polymers can be as large as ${\mathscr{O}}$(20%)^15^, creating a significant uncertainty in analytic theoretical modeling of how chain topology influences polymer solution hydrodynamic properties. Our numerical treatment of these problems does not alleviate the inherent errors in this type of analytic calculation, but we hope that our precise numerical estimates of knot energy functionals will provide impetus for further theoretical efforts aimed at calculating the hydrodynamic properties of polymers in solution. The main problem here is that relatively rare, more extended conformations can give a disproportionate contribution to the hydrodynamic properties so that the properties calculated for “typical” configurations do not describe ensemble average properties. The problem is especially great for flexible polymers when these fluctuation effects are large.

Now that we have characterized many of this basic solution properties of knotted polymers over a range of knot complexities, chain stiffness, and chain length, we plan to extend this work to MD of knotted polymers in the melt state. Since many of the properties of knotted polymers in solution are altered as the knot crossing number *m* is varied, which is similar to prior findings for star polymers having a variable number of arms, we expect to see similar trends relating to the configurational properties of knotted rings and star polymers in the melt state and the properties of the resulting materials when the topological structure of the molecules causes them to have similar average molecular shapes. Simulations of knotted ring and star polymers in the melt state the problems are currently in progress.
